# Rational and Translational Implications of D-Amino Acids for Treatment-Resistant Schizophrenia: From Neurobiology to the Clinics

**DOI:** 10.3390/biom12070909

**Published:** 2022-06-29

**Authors:** Andrea de Bartolomeis, Licia Vellucci, Mark C. Austin, Giuseppe De Simone, Annarita Barone

**Affiliations:** 1 Laboratory of Translational and Molecular Psychiatry and Unit of Treatment-Resistant Psychosis, Section of Psychiatry, Department of Neuroscience, Reproductive Sciences and Dentistry, University of Naples Federico II, 80131 Naples, Italy; liciavellucci2@gmail.com (L.V.); giuseppe27.desimone@gmail.com (G.D.S.); annaritabarone1@gmail.com (A.B.); 2 Clinical Psychopharmacology Program, College of Pharmacy, Idaho State University, Pocatello, ID 83209, USA; markaustin@isu.edu

**Keywords:** antipsychotics, treatment-resistant schizophrenia, NMDA, dopamine, glutamate, D-serine, D-aspartate, D-alanine, D-amino acid oxidase, D-cysteine

## Abstract

Schizophrenia has been conceptualized as a neurodevelopmental disorder with synaptic alterations and aberrant cortical–subcortical connections. Antipsychotics are the mainstay of schizophrenia treatment and nearly all share the common feature of dopamine D2 receptor occupancy, whereas glutamatergic abnormalities are not targeted by the presently available therapies. D-amino acids, acting as N-methyl-D-aspartate receptor (NMDAR) modulators, have emerged in the last few years as a potential augmentation strategy in those cases of schizophrenia that do not respond well to antipsychotics, a condition defined as treatment-resistant schizophrenia (TRS), affecting almost 30–40% of patients, and characterized by serious cognitive deficits and functional impairment. In the present systematic review, we address with a direct and reverse translational perspective the efficacy of D-amino acids, including D-serine, D-aspartate, and D-alanine, in poor responders. The impact of these molecules on the synaptic architecture is also considered in the light of dendritic spine changes reported in schizophrenia and antipsychotics’ effect on postsynaptic density proteins. Moreover, we describe compounds targeting D-amino acid oxidase and D-aspartate oxidase enzymes. Finally, other drugs acting at NMDAR and proxy of D-amino acids function, such as D-cycloserine, sarcosine, and glycine, are considered in the light of the clinical burden of TRS, together with other emerging molecules.

## 1. Introduction

Schizophrenia is a severe psychiatric disorder that affects approximately 0.7% of the world population, striking young adults or adolescents at the onset, and causing significant impairment in social and occupational functioning, seriously impacting the quality of life of patients and their families [1]. Clinically, three main psychopathological domains can be present: positive (hallucinations, delusions, disorganization), negative (flat affect, social withdrawal, anhedonia, avolition), and cognitive (altered executive functions, impaired working memory) symptoms.

Antipsychotics, acting mainly albeit not exclusively through the occupancy of dopamine D2 receptor (D2R), represent the cornerstone of schizophrenia pharmacological treatment and have proven highly effective in managing positive symptoms while being limitedly efficacious on negative and cognitive ones. However, approximately 30% of schizophrenia patients do not respond to two consecutive antipsychotics, each of them used at the highest dose and at least for six weeks of treatment; these patients are defined as treatment-resistant [2,3]. Treatment-resistant schizophrenia (TRS) patients have more severe abnormal functioning [4], cognitive deficits [5,6], and neurological soft signs [7]. The only antipsychotic available for TRS therapy is clozapine [2,8,9,10], a highly efficacious drug whose utilization in clinical practice is undermined by relatively rare but severe adverse events such as agranulocytosis, myocarditis, intestinal hypomotility, and convulsions [11]. Therefore, clozapine is largely underused and frequently introduced very late, when the trajectory of the disease is already advanced [12]. Moreover, it is unrealistic that a complex disorder such as schizophrenia could be treated with just one pharmacological agent tackling all the psychopathological domains of the disease. The complexity of the clinical presentation is mirrored by the complexity of molecular aberrations underpinning the disease neurobiology, driving the concept of schizophrenia as a polygenic and multifactorial disorder characterized by abnormal synaptic plasticity and altered cortical–subcortical connectivity [13,14]. In this framework, there is a significant need for novel pharmacological strategies that take into account preclinical and clinical findings in a direct and reverse translational fashion [15,16]. One of the proposed strategies to counteract poor response to antipsychotics in schizophrenia patients is based on D-amino acids’ utilization or augmentation, and thus several clinical trials have been performed with D-serine, D-alanine, and D-amino oxidase inhibitors to assess the efficacy and tolerability of these novel therapeutic molecules as well as their possible future applicability [17,18,19,20,21,22,23,24,25,26,27,28,29,30]. Finally, a further therapeutic approach in a similar direction is represented by the investigation of a few compounds equipped with D-amino acid-related mechanism of action, such as D-cycloserine, sarcosine [31,32], and glycine, whose preclinical investigation [33] as well as utilization in experimental design have indirectly contributed to expanding our knowledge on D-amino acids potential role in TRS. This review aims at addressing the preclinical and clinical evidence in support of the role of D-amino acids in treatment resistance or poor responder schizophrenia patients. We will tackle the following questions:

(1) How robust is the evidence supporting an alteration of the glutamate system and dopamine-glutamate interaction in TRS?
(2) How and to what extent can D-amino acids dysregulation intercept the glutamatergic abnormalities in TRS?
(3) Do preclinical and clinical findings substantiate the D-amino acid strategy in TRS?
(4) What are the putative innovative future trials and targets?

With these questions in mind, we describe the complex and promising landscape of D-amino acid-based potential interventions in TRS.

## 2. Materials and Methods

**Figure 1:**
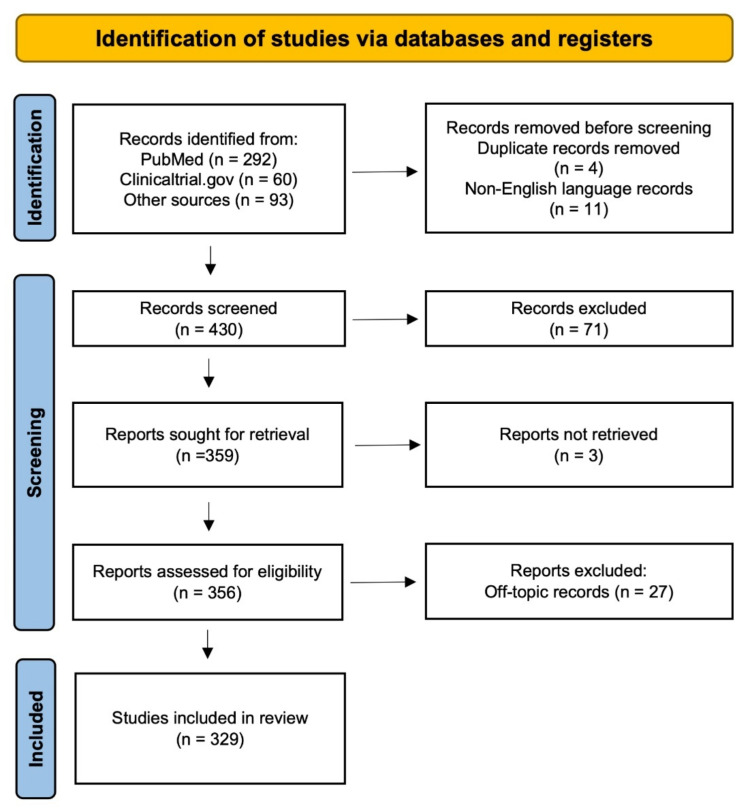
Preferred Reporting Items for Systematic Reviews and Meta-Analyses (PRISMA) flow chart. The diagram details the database searches, the number of abstracts screened, the full-text documents retrieved, and the number of included and excluded studies.

We conducted a systematic review of the literature according to the Preferred Reporting Items for Systematic Reviews and Meta-Analyses (PRISMA) guidelines [34], based on a PubMed/MEDLINE database search from inception up until 5 May 2022, using the following search string: schizophrenia[MeSH Terms] AND ((D-amino acid oxidase[MeSH Terms]) OR (D-aspartic acid[MeSH Terms]) OR (D-serine) OR (D-alanine) OR (D-amino acid)). Searches were also conducted using ClinicalTrials.gov, using the keywords “D-amino acids”, “D-serine”, “D-aspartate”, “DAO inhibitors” or their combinations. Additional records were retrieved by hand-searching the reference lists of included articles. We included in the qualitative synthesis English-written clinical and preclinical studies relevant to the subject, (i) exploring the implications of D-amino acids in the pathophysiology of schizophrenia and TRS; (ii) reporting original data on D-amino acid-centered strategies for the treatment of schizophrenia and refractory psychotic disorders. We did not apply design methodology constraints. After removing duplicates, 430 records were identified. After pilot-testing the study selection, screening for title/abstract and full-text assessment were independently performed by two investigators (AB and LV). Discrepancies were resolved by reaching consensus. The details of the selection process are reported in the PRISMA flow diagram (please see Figure 1). After the screening process, 329 studies were included in the qualitative synthesis.

## 3. The neurobiology of Treatment-Resistant Schizophrenia and Amino Acid-Related Metabolism

Even though the molecular and pharmacological mechanisms of TRS are still elusive, consistent in vivo evidence mainly from multimodal imaging studies has emerged in recent years, shedding light on the putative neurobiological underpinning of this severe medical condition, especially when compared to treatment-responsive schizophrenia.

Among the most replicated imaging findings addressing in vivo neurotransmission in schizophrenia patients, two stand over all: a) the increased release of striatal dopamine after intravenous stimulants (i.e., amphetamines) in schizophrenia patients compared to normal controls as measured by Positron emission tomography (PET) adopting ^11^C-raclopride [35,36,37,38]; and (2) the increased dopamine capacity detected by 3,4-dihydroxy-6-[18F]fluoro-l-phenylalanine (^18^F-DOPA) PET [39]. Both methods and related results strongly support the dopamine hypothesis of schizophrenia [40]. More recently, reduced radioligand binding at D2R, indirectly indicating a high level of dopamine release, has been detected in the thalamus, a region considered a key hub in the cortical–subcortical dopamine–glutamate interaction [41]. However, in patients not responding adequately to antipsychotics and fulfilling the criteria of TRS, a significant increase in striatal dopamine capacity is not detectable, suggesting that in this group of patients the potential neurobiological underpinning of antipsychotics poor response does not lie exclusively on the dopamine cortico-striatal dysregulation [42,43,44].

Among other neurotransmitter systems involved in TRS pathophysiology, the glutamatergic pathway has been the strongest related to disease neurobiology. Glutamate levels have been reported significantly different in multiple brain regions of TRS patients compared to responders and healthy controls, as measured by ^1^H-magnetic resonance spectroscopy (MRS) [44,45,46,47]. In this regard, the anterior cingulate cortex (ACC) has strongly been implicated in TRS pathophysiology, showing an increase in glutamate concentrations not detectable in antipsychotics responder patients [44,45,46,47]. In a multicenter study including 48 responder and 44 non-responder schizophrenia patients, beyond the significant increase in ACC glutamate levels exhibited by the TRS group, the authors measured ^18^F-DOPA striatal uptake and no differences were detected between the two groups [44], supporting the glutamatergic and not dopaminergic involvement in TRS pathophysiology. Moreover, the ACC (glutamate + glutamine)/N-acetyl aspartate (NAA) ratio not only has been found higher in TRS patients in comparison with schizophrenia responsive and healthy control groups but has also been negatively associated with working memory scores measured by the working memory index (WMI) of the Wechsler Adult Intelligence Scales—Fourth Edition (WAIS-IV) [47]. In line with this trend, a decrease in ACC cortical thickness has been related to an increase in ACC glutamate + glutamine levels within TRS and clozapine-resistant patients [48], suggesting that a possible correlation between central glutamate variations and changes in brain structures might provide a fertile background for antipsychotics unresponsiveness. Abnormal ACC glutamate metabolism, poor cognitive performance, and the changes in white matter tracts revealed by MRS have also been reported in early-onset TRS patients, highlighting the possible existence of a predisposing pathological environment present since the beginning of the disease [49]. Further to the clear involvement of ACC, a post hoc analysis conducted in first-episode psychosis (FEP) patients, allowing clinicians to retrospectively define antipsychotic responsiveness or resistance, showed higher glutamate levels in non-responders, positively associated with striatal volume [50]. Again, in the putamen, the total glutamate + glutamine levels have been found increased in TRS patients when compared to first-line responders [51].

To summarize, increasing evidence has been provided in supporting the glutamatergic hypothesis of schizophrenia and even more TRS, with higher glutamate and glutamine levels running in parallel with morphological changes in brain architecture and cognitive decline. The increase in glutamate concentrations has been attributed to N-methyl-D-aspartate receptor (NMDAR) hypofunction, a glutamate ionotropic receptor expressed on the surface of γ-aminobutyric acid (GABA) ergic parvalbumin-positive (PV+) neurons. In fact, poor NMDAR functioning may lead to the disruption of the inhibitory control of the excitatory pyramidal neurons, and thus to the so-called glutamatergic storm [52].

It is noteworthy that glutamate can be affected at multiple molecular levels in refractory schizophrenia [53]: (1) synthesis, which directly involves astrocytes [54]; (2) release [55]; (3) action at postsynaptic [56] and perisynaptic receptors [57]; (4) modulation of postsynaptic density proteins (PSD) [58] and intracellular signaling cascades [59]; (5) reuptake by glial cells through excitatory amino acid transporters (EAATs) [60]. In this framework, D-amino acids and related enzymes may directly or indirectly influence these processes.

Considering the discussed role of glutamate in general schizophrenia pathophysiology and, even more, in TRS, it is not surprising that D-amino acids have been envisioned as a potential pharmacological intervention for strengthening the response to antipsychotics in those patients that do not show improvement with the available treatments [61]. Of interest, one of the earliest reports assessing D-amino acids’ involvement in TRS pathophysiology demonstrated a difference in D-serine plasma levels between TRS and healthy controls, as well as an increase in glycine and D-serine concentrations after clozapine treatment [18], despite the limitation of the “peripheral” detection, which may partially mirror central measures.

It has been proposed that clozapine, the cornerstone of treatment in resistant schizophrenia, may exert its unique antipsychotic effect by directly tuning glutamatergic signaling rather than merely blocking dopaminergic receptors. In fact, neither typical antipsychotics nor D1R agonists can mimic its molecular effects in restoring the glutamatergic pathways [62]. Several lines of evidence emphasized the glutamatergic mode of action of clozapine [63,64], paving the way for the use of modulators of glutamatergic transmission, namely D-amino acids, in the treatment of TRS [62].

The D-amino acid-centered pharmacological strategies may intercept multiple steps of glutamate signaling, directly targeting NMDAR function or increasing D-serine and D-aspartate synaptic concentration via inhibition of D-amino acid oxidase (DAO) and D-aspartate oxidase (DASPO), respectively, both involved in the regulation of NMDAR function [65,66].

## 4. Dopamine–Glutamate Interaction and the Role of D-Amino Acids

The dopamine hypothesis of schizophrenia has been one of the most enduring assumptions on the origin of the disease, based on the evidence of antipsychotics acting as D2R blockers, and considering the striatal dopamine hyperactivity as responsible for the occurrence of positive symptoms [35,67,68]. Further evidence has been derived from neuroimaging studies showing an increase in striatal dopamine levels after acute amphetamine administration in schizophrenia patients compared to healthy controls [35,37]. On the other hand, clinical observations that acute and repetitive exposure to phencyclidine (PCP), ketamine, and other NMDAR non-competitive antagonists could similarly trigger psychotic-like symptoms in healthy subjects or exacerbate psychotic symptoms in schizophrenia patients have fueled the alternative but not mutually exclusive glutamate hypothesis of schizophrenia [69,70]. This hypothesis has been corroborated by multiple in vitro and in vivo experimental findings [71,72,73]. For instance, in vivo PET imaging studies have shown that NMDAR antagonists, similarly to stimulants, are able to reduce striatal ^11^C-raclopride D2R binding, suggesting an increase in striatal dopamine release in humans [74,75,76]. Moreover, NMDAR antagonists have been found to induce impairment in executive functions, mimicking cognitive symptoms of schizophrenia, thus supporting the involvement of glutamatergic transmission in the pathophysiology of the disorders [77,78,79,80,81]. The rise of the glutamate hypothesis of schizophrenia has not eclipsed the well-established dopamine theory of schizophrenia, therefore originating a more complex interpretation of the disease based on dopamine-glutamate interaction in which glutamate and dopamine pathways in schizophrenia pathophysiology are converging at multiple levels and with reciprocal influence [82,83].

Furthermore, several lines of evidence demonstrated that NMDAR hypofunction affects corticolimbic GABAergic PV+ interneurons, reducing their excitability with a subsequent loss of their inhibitory action on glutamatergic intracortical neurons controlling downstream midbrain dopaminergic neurons [84]. In turn, dopaminergic firing increases [85,86,87] and contributes to specific schizophrenia-like phenotypes, as shown in transgenic mice, supporting the GABAergic hypothesis of schizophrenia [86,88]. Nikolaus and colleagues presented a series of experiments exploring the effects of agonists and antagonists at both GABA_A_ receptors and NMDARs on dopamine controls in mesolimbic and mesostriatal pathways. They observed that the GABA_A_ receptor agonist muscimol and the NMDAR antagonist amantadine exert similar actions on regional dopamine release, supporting the hypothesis of triple glutamate—GABA—dopamine interaction underlying the dysfunctions observed in schizophrenia [89,90].

Beyond the well-known effect on the dopaminergic pathway, stimulants such as cocaine have been seen to variously modulate the glutamatergic system, participating in the cross-talk between the two neurotransmitters. Specifically, after cocaine withdrawal, a reduction in glutamate concentration has been detected in the nucleus accumbens of rodents, associated with an increase in dendritic spine head diameter and α-amino-3-hydroxy-5-methyl-4-isoxazolepropionic acid receptor (AMPAR) surface expression, hence raising the AMPAR/NMDAR ratio [91,92]. Furthermore, the dopamine D1 receptor (D1R) stimulation has been shown to enhance glutamatergic neurotransmission, increasing NMDAR phosphorylation and activity via the induction of the downstream protein kinase A (PKA)/dopamine and cAMP-regulated phosphoprotein-32 (DARPP-32)/protein phosphatase 1 (PP1) pathway [93].

The integration of dopaminergic and glutamatergic signals occurs in the PSD, a functional interface acting as a bridge between postsynaptic receptor structures and intracellular downstream transduction pathways [58,94,95,96]. The PSD has been described as a thickening of the glutamatergic postsynaptic terminals, composed of glutamate receptors, ion channels, scaffolding proteins, adaptors, cytoskeletal proteins, and modulators of signaling processes [97,98,99]. Several lines of evidence point to PSD proteins as key molecules implicated in schizophrenia pathophysiology and possibly in its pharmacological treatment [100,101,102,103,104].

Multiple reports have demonstrated that the striatal induction of immediate early genes (IEG), coding for PSD proteins, depends on D1R and its intracellular signaling cascade [105,106,107,108,109]. Nonetheless, the effects of pro-dopaminergic agents such as amphetamines and cocaine on IEG induction may be inhibited by NMDAR antagonists, suggesting the presence of a dopamine–glutamate interaction at the level of striatal circuits or at least at the intracellular level [110]. Moreover, the striatal IEG expression may be differentially modulated according to the specific NMDAR subunit expressed [111]. In fact, the blockade of GluN2A-containing-NMDAR has been associated with a reduction in *c-Fos* and *Zif-268* expression, which otherwise increased after D1R agonist administration [111]. Contrarily, the antagonism at GluN2B-containing-NMDAR has an opposite effect on gene expression, suggesting different regulation of striatal function depending on the specific subunit composition [111].

Through the expression of several PSD proteins, dopaminergic perturbations have been accounted for regulating the shape and growth of dendritic spines and thus the synaptic NMDAR functioning [112]. In particular, the activation of D1Rs but not D2Rs or NMDARs has been found to induce the transcription of Homer1a, a component and regulator of PSD, in the prefrontal cortex (PFC), nucleus accumbens, and ventral tegmental area (VTA) [113]. The elevated intracellular levels of Homer1a protein may lead to: (i) changes in dendritic spines and axons via altered PSD proteins recruitment (such as PSD-95); (ii) disruption of protein complexes and subsequent translocation of metabotropic glutamate receptors (mGluRs) to the membrane surface; (iii) increased calcium entry via transient receptor potential (TRP) calcium channels; (iv) decoupling of mGluR5 and extracellular signal-regulated kinase (ERK) signaling, exerting neuroprotective properties [113,114,115,116,117,118,119,120,121,122,123,124,125,126,127,128,129,130,131,132,133,134,135]. Moreover, Homer1a overexpression has been found to blunt the glutamate response associated with acute cocaine administration [136,137].

With regard to D1R-NMDAR cross-talk converging on PKA/DARPP-32/PP1 pathway, PSD-95 overexpression has been found to disrupt physical interactions by interposing between dopamine and glutamate receptors [138]. On the other hand, lack of PSD-95, as shown in mutant mice, is responsible for the overactivation of both D1R and NMDAR, increasing the susceptibility to NMDAR-mediated excitotoxicity, with subsequent neuronal damage [139].

In this framework, D-amino acids including D-serine may exert a relevant role in dopamine and glutamate interplay, enhancing NMDAR-dependent excitatory postsynaptic currents (EPSCs), and modulating the downstream dopamine neurons’ excitability, indicating a putative involvement in the neurobiological core of schizophrenia [140]. Of interest, D-serine has shown the capability to modulate NMDAR firing, mainly acting as a receptor co-agonist at synaptic sites, in contrast to glycine, exerting its role at the extrasynaptic level [141]. Moreover, it has been observed that the genetic deletion of the DASPO encoding gene has a relevant impact on the expression of PSD proteins [142], highlighting potential implications of D-amino acids for the architecture of the dendritic spine that relies on PSD composition. Indeed, considering PSD, a molecular hub integrating dopamine and glutamate interaction, the D-amino acid impact on this structure is even more appealing as a therapeutic strategy for schizophrenia.

Even a dopaminergic influence on D-serine availability has been suggested, with different roles exerted by D1R and D3R on D-amino acid levels. In particular, tonic spontaneous dopamine release not only enhances NMDAR firing through D1-type receptors but also increases D-serine concentration in PFC, ameliorating cognitive performances in animal models [143]. On the contrary, high phasic dopamine release activates D2-type receptors, which reduce both D-serine levels and NMDAR activation [143].

D-serine has been involved also in GABA–glutamate interplay, being synthesized in striatal GABAergic neurons, serving as a modulator of NMDAR excitability at postsynaptic GABAergic dendrites, which receive glutamatergic afferents, thus limiting the diffusion of glutamatergic inputs from the brain cortex to other structures [144].

Therefore, the relevance of dopamine–glutamate interplay for understanding the neurobiology of schizophrenia, as well as the ability of D-amino acids to interfere with these complex interactions, have raised keen interest in developing novel D-amino acid-based therapeutic strategies.

## 5. D-Amino Acids as an Innovative Potential Therapeutic Approach to Mitigate NMDAR Hypofunction in Schizophrenia

In the last two decades, the theories about neurotransmission dysfunction in schizophrenia shifted from a classical “dopamine-centric” hypothesis toward a more complex neurotransmitter imbalance, including mainly glutamatergic, serotonergic, GABAergic, and peptidergic transmission [75,77,78,79,80,81,86,88].

Glutamate is the major excitatory neurotransmitter in the central nervous system (CNS), and it is involved in various processes, such as memory, learning, and brain development, through the action on two fundamental types of postsynaptic receptors, namely ionotropic (i.e., NMDAR, AMPAR, and kainate) and metabotropic ones (divided into Groups I, II, and III) [145]. The activation of NMDAR requires its endogenous agonist glutamate and the co-agonist glycine or D-serine to be bound to specific binding sites on the receptor complex [145,146]. The hypofunction of NMDAR has been proposed in schizophrenia upon observing that PCP and its analogues, such as ketamine and MK-801, non-competitive antagonists at NMDAR, can induce a wide range of psychotic symptoms in healthy individuals, not only positive but also negative and cognitive ones, recapitulating the most relevant features of the disease [77]. Particularly, acute and chronic PCP abuse can replicate a schizophrenia-like clinical picture more closely than amphetamines, which are unlikely to mimic negative symptoms and cognitive deficits [79,147,148].

In this framework, NMDAR dysfunction in schizophrenia represents a convergence point of dopaminergic, glutamatergic, and GABAergic alterations, as well as the final common pathway leading from the pathophysiology to the symptom progression [149].

Of interest, the exacerbation of symptoms in schizophrenia patients induced by NMDAR antagonists is only partially relieved by canonical antipsychotics, reacting better at the second-generation neuroleptics having a broader receptor profile, which allows interaction with different neurotransmitter systems. In this context, the possibility of minimizing the hypofunction of NMDAR through the use of D-amino acids acting as co-agonists at this site opens new attractive scenarios.

Psychotropic modulation of glutamate neurotransmission may represent potential monotherapy or add-on treatment strategy for TRS [150]. In particular, D-amino acids augmenting the NMDAR-related transmission may be used in schizophrenia to ameliorate the clinical response to conventional medications.

In the following sections, we will extensively review the preclinical and clinical evidence supporting research attention to D-amino acids and their therapeutic potential in refractory psychiatric disorders.

### 5.1. NMDAR Co-Agonists

NMDARs in CNS are heteromeric protein complexes composed of at least one GluN1 subunit together with different combinations of GluN2 and/or GluN3 [151]. Alternative splicing of the *GRIN1* gene leads to eight GluN1 isoforms, which are required for channel function. The association of GluN1 subunits with different variants of GluN2 (GluN2A-D) and GluN3 (GluN3A and GluN3B) produces a variety of NMDARs with peculiar biophysical and pharmacological properties and specific expression patterns in development and adulthood [152,153]. The dynamic regulation of NMDAR’s activity is also achieved through post-translational modifications, such as glycosylation, phosphorylation, and ubiquitination [154].

**Figure 2:**
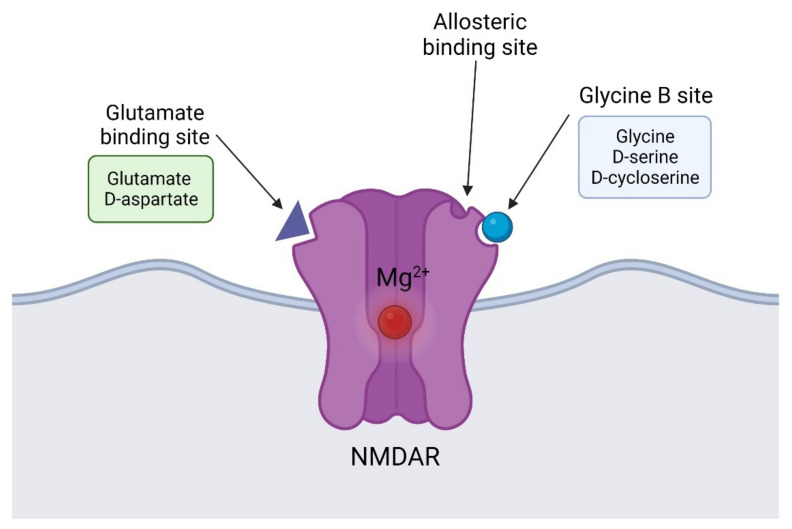
NMDAR structures and binding sites. Whereas glutamate binds to a dedicated glutamate-binding site, the co-agonists D-serine and glycine bind to the so-called “glycine B site”. D-cycloserine acts as a partial agonist at this site. NMDAR activation requires the concomitant binding of glutamate and co-agonists. NMDAR: N-Methyl-D-aspartate receptors; Mg^2+^: magnesium. Created with BioRender.com (accessed on 14 June 2022).

At resting membrane potential, the pore of the NMDAR channel is blocked by Mg^2+^, a block that can be removed upon depolarization by neighboring AMPARs. Aside from the glutamate/NMDA recognition site, GluN1 exhibits a second site for glycine/D-serine (named glycine modulatory site or glycine B site) (Figure 2) [155], and both must be occupied for channel activation. Once finally opened, the NMDAR channel allows for an influx of calcium ions, which activates an intracellular signaling cascade that can ultimately involve gene transcription and synaptic plasticity. NMDAR recruitment during high presynaptic glutamatergic activity results in a permanent increase in synaptic efficacy known as long-term potentiation (LTP) [156]. The persistent activation of NMDAR can cause the sprouting of postsynaptic spines and also have trophic effects on postsynaptic neurons [157], but, on the other hand, an excessive activation can produce oxidative stress and subsequent neuronal death [158]. Indeed, direct agonists at NMDAR are known to be neurotoxic by providing excitotoxicity [159]. Thus, in order to mitigate the NMDAR hypofunction and avoid the excitotoxic consequences of direct receptor activation, current strategies have been carried out on agonists at the glycine B site of the NMDAR complex, using full agonists including D-serine, D-alanine, and glycine, as well as partial agonists such as D-cycloserine.

### 5.2. D-Serine

**Figure 3:**
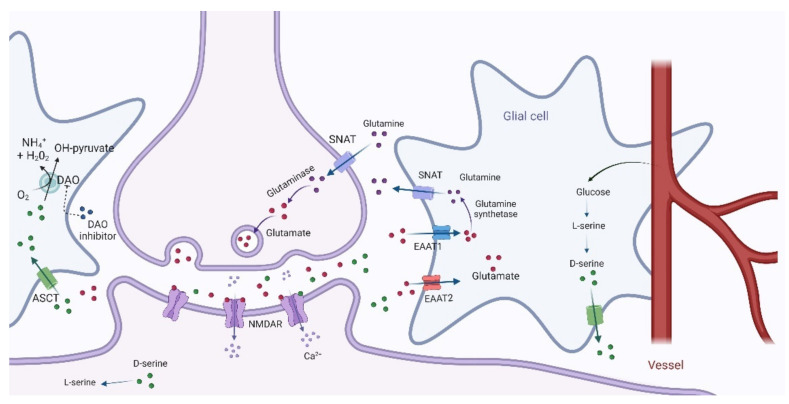
D-serine is synthesized in astrocytes by serine racemase, an enzyme that converts L-serine into D-serine. D-serine acts as a co-agonist at synaptic NMDARs, whereas its reuptake is performed by the neutral amino acid transporters Alanine-serine-cysteine-threonine (ASCT) 1 and 2. D-amino acid oxidase is responsible for D-serine degradation in glial cells. ASCT: Alanine-serine-cysteine-threonine transporter; EEAT1: Excitatory amino acid transporter 1; EEAT2: Excitatory amino acid transporter 2; SNAT: Sodium-coupled neutral amino acid transporter; NMDAR: N-Methyl-D-aspartate receptors; DAO: D-amino acid oxidase. Created with BioRender.com (accessed on 14 June 2022).

Serine is one of the naturally occurring proteinogenic amino acids synthesized in the human body from other metabolites, including glycine. D-serine is synthesized in the brain by serine racemase from L-serine (Figure 3), whose biosynthesis controls its levels [160]. It acts as an endogenous ligand at the glycine B site of NMDAR, playing a central role in mediating NMDAR signaling and plasticity [161]. Recently, abnormalities in the D-serine pathway have been found to suggest a significant contribution to glutamatergic dysfunctions.

As mentioned above, NMDAR activation requires the concomitant binding of glutamate and at least one of glycine or D-serine. However, D-serine was found to be more effective than glycine in increasing glutamatergic neurotransmission [162,163,164,165,166,167,168].

In particular, previous studies have shown that the effective dose required to activate NMDAR was lower for D-serine as compared to glycine, probably due to aromatic residues affecting binding kinetics at the glycine binding site [166,169]. Moreover, immunohistochemical studies have shown that in the telencephalon and developing cerebellum D-serine is expressed in close proximity to NMDARs, whereas the distribution of glycine overlaps the expression of NMDAR in the brainstem, olfactory bulb, and adult cerebellum [170], pointing to D-serine as the major endogenous ligand at the glycine B site at least in the forebrain. In addition, in vivo microdialysis revealed that the extracellular content of free endogenous D-serine was approximately 2.5 times higher than that of glycine in the striatum while being markedly lower in the cerebellum [171].

Basu and colleagues demonstrated that a lack of D-serine may be crucial in the pathophysiology of schizophrenia as observed in a murine model of constitutive D-serine deficiency [172]. They found that mutant mice lacking the capacity to endogenously produce D-serine presented significant alterations in glutamatergic transmission with a subsequent serious deficit in spatial memory and synaptic plasticity, thus reproducing cognitive impairments associated with the schizophrenic endophenotype [172]. Moreover, mutant mice carrying mutations in serine racemase, resulting in a complete loss of enzyme activity, exhibit dramatically reduced D-serine levels and a number of psychotic traits, a significant reduction in the density of inhibitory synapses in the hippocampus [173], and impairments in sensorimotor gating, sociability and spatial discrimination of stimuli [174,175]. Persistent latent inhibition observed in mutant mice carrying a point mutation in NMDAR co-agonist site was effectively reversed by D-serine, which enhances NMDAR glycine B site function, as well as the atypical antipsychotic clozapine [176], whose mechanism of action could involve glial and neuronal exocytosis of D-serine in medial PFC [177].

The relevance of the D-serine pathway in regulating glutamatergic tone is also suggested by in vitro tests indicating the ability of D-serine to enhance NMDAR currents in the hippocampus and striatum [164,178,179]. According to this line of research, increased hippocampal activation after D-serine administration was also corroborated by functional magnetic resonance imaging (fMRI) findings [180].

Several abnormalities in the levels of D-serine and enzymes that modulate D-serine availability were checked in patients suffering from schizophrenia [181]. For instance, a significant reduction in D-serine levels has been found in cerebrospinal fluid (CSF) [182,183] and the serum of patients affected by schizophrenia [184,185], despite discrepancies [186,187]. Furthermore, Ono and colleagues [188] reported a significant elevation in DAO expression in choroid plexus epithelial cells, hypothesizing a subsequent reduction in D-serine cerebrospinal levels in schizophrenia. In a cross-sectional study, Ozeki and colleagues observed an increase in the activity of phosphoserine phosphatase, a rate-limiting enzyme in L-serine synthesis, in peripheral blood mononuclear cells from schizophrenia patients, and a subsequent rise in plasma L-serine accompanied by a reduction in D-serine levels [189].

With regard to the brain concentration of D-serine and related enzymes, as revealed by a machine learning analysis conducted in a post-mortem schizophrenia brain, D-serine resulted to be among the molecules mostly contributing to discriminating schizophrenia from controls [190]. Several studies have also identified abnormalities in DAO expression and activity in the cerebellum [188,190,191], hippocampus [192], and cortex [193]. Verrall et al. detected an elevation in DAO mRNA in the cerebellum of patients, consistent with increased D-serine degradation, whereas serine racemase levels increased in dorsolateral PFC [194], probably reflecting an attempted compensatory mechanism for adapting to the reduction in cortical D-serine. However, given the physiological absence of DAO in the forebrain, which is enriched with D-serine, serine racemase in these areas is known to perform also α,β-elimination reaction with both L-serine and D-serine, contributing to the control of D-serine levels [195,196]. Genetic studies evaluated the association of schizophrenia phenotype with polymorphisms in genes involved in D-serine metabolism. For instance, protein interacting with C kinase (PICK1), a protein required for serine racemase functioning [197], has been found to be altered in “disorganized” schizophrenia, as reported in a case–control study enrolling Japanese subjects [198]. Several studies indicate an association between DAO gene, its potential regulator G72, and schizophrenia [199,200,201,202,203,204]. Of interest, Disrupted-In-Schizophrenia-1 (DISC-1), which has been repeatedly associated with schizophrenia [205], has been found to bind to and stabilize serine racemase. In a preclinical study, it has been demonstrated that mutant DISC-1 fails to bind serine racemase, resulting in a reduction in D-serine levels with subsequent behavioral abnormalities consistent with hypofunction of NMDAR [206]. Conversely, the administration of D-serine in rats seems to have a sex-specific effect, inducing an increase in DISC-1 levels in dorsolateral PFC of female rats and a decrease in males [207]. In the same study, the authors showed that D-serine reduced the expression, in male rats, of nitric oxide synthase 1 adaptor protein (NOS1AP), which has been found overexpressed in the cortex of patients with schizophrenia [207]. Based on the evidence of NOS1AP gene involvement in schizophrenia [208], the relevance for dendrite branching [209], and its role in mediating NMDAR-PSD-95 signaling, further studies suggested that its overexpression in dorsolateral PFC of patients may result in disruption of NMDAR functions [210,211,212]. Similar behavioral abnormalities in adulthood are elicited also by neonatal injection of a selective inhibitor of the enzyme [213].

Although neither conventional nor atypical antipsychotics directly affect brain levels of D-serine [19,194,207,214], it has been argued that amelioration of schizophrenia symptoms is related to elevation in plasma D-serine levels [215,216]. Moreover, several preclinical and clinical studies have shown an association between D-serine availability and cognitive and negative symptoms in schizophrenia [217,218,219]. Indeed, in an animal model of anhedonia with a deficit in sucrose consumption induced by MK-801, acute D-serine administration at 1280 mg/kg is responsible for attenuating MK-801 effects similarly to clozapine, but not haloperidol administration [218]. Furthermore, the systemic administration of D-serine (50 mg/kg/day) is responsible for improved performance in tasks linked to recognition learning and working memory [220].

In a recent preclinical study, the involvement of D-serine in the fine-tuning modulation of dopamine-glutamate cross-talk through D1R and D3R was demonstrated. Of interest, the activation of D1R and D3R has been found to regulate the extracellular levels of D-serine by exerting, respectively, a facilitatory and inhibitory influence on D-serine availability, and thus on NMDAR activation. Thus, dopamine may exert a dual effect on glutamatergic transmission due to, among other mechanisms, the mediation of D-serine, which could represent a nodal role in the dopamine–glutamate interface [143]. Furthermore, D-serine seems to potentiate PFC-dependent cognitive processes by D3R blockade [143].

In the light of this evidence, D-serine has been considered a promising strategy for the rational design of novel pharmacological approaches for treating schizophrenia. Clinical trials testing D-serine administration in schizophrenia subjects are discussed below.

#### Clinical Reports of D-Serine Efficacy in Treating Schizophrenia Patients Not Responding to Antipsychotics

D-serine has been tested in several randomized double-blind placebo-controlled trials [221,222,223,224,225,226,227,228] as an adjunctive medication in patients receiving risperidone [224], olanzapine [222], clozapine [227], other antipsychotics, or placebo (Table 1). Negative results were obtained with the clozapine combination [227], probably due to the clozapine mechanism of action already involving the modulation of NMDAR functions. A lack of improvement in the Positive and Negative Syndrome Scale (PANSS) total score, positive and negative subscales, and the scale for the assessment of negative symptoms (SANS) in other trials have been ascribed to the dose used (30 mg/kg or 2g/day instead of 60 mg/kg) [221,228] or the confounder of psychotic exacerbation in trials enrolling acutely ill patients instead of chronic ones [224]. However, a recent systematic review and meta-analysis including the above-cited studies demonstrated the effectiveness of D-serine in reducing the total SANS score and the negative subscale of PANSS [229].

Underlining the relevance of the dose administered, also in the light of the extensive and rapid metabolism via DAO, responsible for a reduced bioavailability of the drug [61], it should be noted that Kantrowitz et al. performed a dose-escalation study demonstrating large effect-size improvements in psychotic symptoms both at 60 mg/kg and 120 mg/kg [230]. The same group reported a decrease in the PANSS total score and improvements in auditory mismatch negativity in patients suffering from schizophrenia and schizoaffective disorder after administration of 60 mg/kg/day of D-serine as an add-on treatment to antipsychotics [223].

Taken together, these findings may suggest that 60 mg/kg may represent an adequate dosage to exert a full antipsychotic response, but the optimal dose required remains to be elucidated. Further research exploring the effects of higher doses of D-serine is underway [231]. However, since the catabolism of high doses of D-serine has been shown to produce putative cytotoxic metabolites for glial cells in vitro [232,233] and cause acute tubular necrosis in rats [234], inhibitors of the DAO function have recently been proposed as a safer alternative to D-serine [235,236,237,238].

Although so far considered a promising strategy to treat refractory schizophrenia patients, a study testing D-serine effects in recent-onset schizophrenia is ongoing [239].

### 5.3. D-Alanine

D-alanine is one of the D-amino acids naturally occurring in the mammalian tissues, present in the human CNS at a concentration estimated to be approximately 12.3 nmol/g in white matter and 9.5 nmol/g in gray matter [240,241,242].

Studies conducted in rats showed that D-alanine concentration reaches a peak at 6 weeks and decreases significantly with age [243]. Moreover, the D-alanine amount slowly declines during the night while increasing during the daytime [243]. Mammals are not capable of synthesizing D-alanine [244], which is primarily derived from the diet, as opposed to D-serine that is biosynthesized by serine racemase [245]. In the mammalian brain, D-alanine plays as a selective and potent co-agonist at NMDARs glycine B site [246] and, similarly to D-serine, it is metabolized by DAO [247]. However, it has been observed that D-alanine may also be directly excreted by the kidneys without being metabolized by DAO [248]. In fact, the co-administration of the DAO inhibitor sodium benzoate and D-alanine did not change D-alanine concentration in CSF in comparison to D-alanine alone, suggesting that this enzyme is not crucial in the regulation of the D-alanine availability [249].

Consistent with glutamatergic dysfunction in schizophrenia, D-alanine administration in animal models is responsible for inhibiting PCP-induced locomotor activity [250], and methamphetamine-induced hyperactivity [251]. Moreover, intracortical infusion of D-alanine was able to attenuate PCP-induced accelerating effects on prefrontal dopamine metabolism [252].

The role of D-alanine in the neurobiology of schizophrenia requires further investigations but increasing evidence has highlighted differences in plasma levels of this amino acid in patients compared to healthy controls. In this regard, Hatano and colleagues demonstrated that D-alanine plasma levels in patients diagnosed with schizophrenia increased significantly from hospital admission, due to higher severity of symptoms, to discharge, positively correlating with clinical improvements [253].

Based on these clinical and preclinical findings, D-alanine was also evaluated in clinical studies (Table 1) testing its efficacy in the treatment of schizophrenia as an add-on to other antipsychotics.

A 6-week double-blind placebo-controlled study reported an improvement in schizophrenia symptoms (based on PANSS positive and Cognitive, and SANS score) after D-alanine administration at 100 mg/kg/day in conjunction with antipsychotics [27].

### 5.4. D-Aspartate

Along with D-serine, D-aspartate is probably the only other D-amino acid synthesized endogenously by a specific aspartate racemase [254]. In the animal brain, D-aspartate has been detected in hippocampal and frontal cortex neurons, mainly concentrated at the distal tip of axons as well as in neuroendocrine cells and CSF [255,256,257,258]. As an excitatory neurotransmitter, D-aspartate is stored in synaptic vesicles and secretory granules at the axon terminal and is released via a calcium-dependent exocytotic mechanism [259,260]. The transporter system responsible for D-aspartate traffic toward vesicles and its reuptake is yet to be identified but may involve glutamate transporters [261].

D-aspartate exhibits a high time-dependent variability in brain expression, switching from a peak in the gestation period to a dramatic reduction in postnatal life [256,262], running in parallel to the progressive increase in DASPO levels, its catabolizing enzyme [259,263,264,265].

Unlike D-serine, which potentiates NMDAR transmission by acting at the modulatory glycine B site on the GluN1 subunit, D-aspartate stimulates postsynaptic NMDARs by directly binding to the glutamate site on the GluN2 subunit [266,267]. Whereas the spatiotemporal distribution of D-serine seems to closely mimic that of NMDARs, the rise and decline in D-aspartate levels, independently of NMDAR expression, suggests its early contribution to the critical period of CNS maturation [262] and a limited function in the following stages of development.

DASPO is a peroxisomal flavoenzyme primarily expressed in neuronal cells, encoded by the *Ddo* gene, and involved in the oxidative deamination of acidic D-amino acids, including D-aspartate and D-glutamate, with the following α-keto acid, ammonium, and hydrogen peroxide (H_2_O_2_) production [24,268,269]. Even though DAO and DASPO may share a similar molecular structure and a common ancestral origin, the two enzymes diverge for several properties, showing different kinetic binding dynamics, flavin adenine dinucleotide (FAD) affinity, and substrate specificity, with a higher selectivity for neutral and basic or acidic D-amino acids, respectively [24,270]. In this regard, DASPO has shown a 10-fold higher specificity for D-aspartate compared with DAO specificity for D-serine [271,272].

Given the selective D-aspartate time-dependent expression pattern, the availability of *Ddo* knock-out murine models (*Ddo*^−/−^) has generated great interest in the scientific community, allowing clinicians to investigate the effect of DASPO and related metabolites during the whole neurodevelopmental process, thus providing a reliable translational model for mammalian. Moreover, the persistent accumulation of D-aspartate observed in *Ddo*^−/−^ mice has suggested that DASPO is the unique or at least the major enzyme capable of degrading D-aspartate. Therefore, high enzymatic efficiency might account for enhanced D-aspartate catabolism, responsible for impairments in early NMDAR-related critical processes [22].

Both oral administration of D-aspartate and genetic deletion of *Ddo* have been found to enhance LTP in mice hippocampus and induce several neuroplasticity modifications, including increased basal brain metabolic activity, dendritic arborization, and spine density [273,274]. Moreover, oral D-aspartate supplementation but not *Ddo* inactivation has been associated with a significant reduction in cognitive flexibility, interpreted by the authors as the result of LTP saturation [273]. Another plausible explanation for cognitive deterioration may rely on the putative burst in H_2_O_2_ production resulting from DASPO enhancement in the D-aspartate treated group, which promotes the establishment of a neuroinflammatory environment.

Since NMDAR expression has not been found altered in several *Ddo* knock-out models, it has been proposed that the D-aspartate facilitation of LTP induction and maintenance may be exerted by enhancing the sensitivity of NMDAR to endogenous glutamate. Of interest, the effects of D-aspartate administration on glutamatergic transmission are not completely reversed by the NMDAR antagonist MK-801, suggesting that D-aspartate action may be mediated, at least in part, by other receptor complexes, including metabotropic mGluR5 and presynaptic AMPARs [275,276,277].

In light of the glutamatergic hypothesis of schizophrenia centered on NMDAR hypofunction, D-aspartate’s ability to influence NMDAR-dependent postsynaptic currents has gained much attention, and thus, several studies have focused on a possible link with psychotic disorders [142,278,279]. Moreover, the possibility to modulate NMDAR and mGluR5 transmission without producing severe excitotoxic effects has turned the therapeutic focus to D-aspartate and DASPO inhibitors. Accordingly, post-mortem studies revealed that D-aspartate content was reduced by 30-40% in PFC and striatum of schizophrenia patients compared with healthy individuals, due to a concomitant 25% increase in DASPO cortical activity [278,280]. A recent machine learning approach confirmed that, among the molecules of glutamatergic pathway highly predictive of schizophrenia in post-mortem dorsolateral PFC, there were D-serine, D-aspartate/total aspartate ratio, and other postsynaptic proteins such as PSD-95, exhibiting a complex non-linear relationship with the probability of developing the disorder [190]. Errico and colleagues demonstrated also that D-aspartate elevation attenuated the disruptive effects of stimulants and MK-801 on pre-pulse inhibition, a measure of sensorimotor gating generally found abnormal in schizophrenia [281].

Furthermore, several lines of evidence point to the involvement of the D-aspartate pathway in the mechanism of action of well-known antipsychotic agents. For instance, the atypical antipsychotic olanzapine has been reported to exert its pharmacological effects through the inhibition of the DASPO enzyme [282]. Furthermore, both clozapine and olanzapine increase the L-glutamate efflux in PFC as observed after D-aspartate administration [282]. Therefore, the increase in DASPO activity resulting from post-mortem findings should not be ascribed to the action of antipsychotics—which, on the contrary, can even inhibit enzyme activity—but should be considered a primitive feature of the disease.

Intriguingly, whereas D-aspartate increase in *Ddo*^−/−^ mutant mice has been found to improve memory and LTP in young rodents, it accelerated the age-related decline in cognitive functioning [283]. This biphasic effect on cognition and synaptic plasticity may be explained by striking age-dependent abnormalities induced in the phosphorylation status of ERK1/2 [283]. In fact, the chronic dysregulation of D-aspartate signaling might account for both the enhancement and the worsening of cognitive abilities, mirrored at the molecular level by the initial pro-survival effects of synaptic NMDAR activity and the subsequent harmful effects of excessive NMDAR stimulation [284]. This dual action may account for conflicting results of D-aspartate-centered strategies in ameliorating cognitive symptoms [257,273]. In this context, the physiological postnatal arise in DASPO expression may be necessary to terminate D-aspartate signaling no longer required in adulthood, thus preventing the detrimental neurodegeneration triggered by excessive NMDAR stimulation.

However, it should be noted that D-aspartate is currently approved and marketed as a dietary supplement without producing toxic effects in humans [285,286].

## 6. D-Amino Acid Oxidase

DAO is an enzyme involved in D-serine and other D-amino acids catabolism via a process of oxidative deamination leading to the production of α-keto acids, ammonium, and H_2_O_2_ [287]. As discussed above, DAO and DASPO share a common structure and probably the same origin but exhibit a different substrate specificity and a dissimilar brain expression pattern.

Whilst DAO activity has been detected in human and other mammalian brains, kidneys, and livers [288], the enzyme has not been found expressed in mouse liver [289]. Therefore, given the limitations of species-to-species translatability, caution should be paid in interpreting the results of preclinical studies assessing the efficacy and tolerability of DAO inhibitors in murine models.

Several studies have investigated the oxidase localization in the CNS aiming at identifying the exact enzyme site of action and multiple regional patterns of DAO expression. At the subcellular level, the oxidase was detected in microperoxisomes of both kidney and brain tissues [290]. Moreover, DAO has been traced almost exclusively within type I and type II astrocytes and other neuron supporting cells such as Bergmann glial cells in the cerebellum [290,291,292], while pyramidal neurons have been identified as the primary enzyme localization in other brain regions, including the hippocampus, PFC, and substantia nigra [194]. Although ubiquitously present, higher amounts of DAO mRNA levels have been detected in the cerebellum than in other areas [291,293,294], as opposed to enzyme protein levels, which are mostly expressed in the amygdala, striatum, and PFC and less in the cerebellum [293]. A plausible explanation for different brain patterns exhibited by DAO mRNA and protein expression may include post-transcriptional modifications via microRNA regulation [293].

Considering the evidence of D-serine-mediated NMDAR modulation and the relevant role in D-amino acid metabolism exerted by the oxidase, it is not surprising the growing interest in DAO as a possible neurobiological target for treating psychotic disorders. The first evidence linking DAO with schizophrenia came from a genetic association study seeking disease-related single nucleotide polymorphism (SNPs) in a 5-Mb segment of the chromosome 13q34 [203]. The authors were able to identify G72 as a potential gene involved in schizophrenia pathophysiology and discovered that the gene encoded for a DAO binding protein, known as DAO activator (DAOA), capable of enhancing the enzyme-induced D-serine oxidation [203]. Thus, an analysis of the DAO gene was performed, and four intronic SNPs were found to be associated with schizophrenia in a French-Canadian sample [203]. Noteworthy, as demonstrated by *post-mortem* studies, the oxidase activity and expression increased bilaterally in the hippocampus and cerebellum of patients suffering from schizophrenia than in healthy controls [183,191,192]. Further, the ratio between serine racemase and DAO protein levels was shown to be significantly lower in the disease group, possibly explaining the decrease in D-serine concentrations observed in the CSF of the affected population [183,191,192]. Afterwards, several experiments have been conducted in murine models to elucidate the putative impact of DAO impairment on cognitive functions. In this regard, DAO knockout mice exhibited better spatial and non-spatial short-term memory performance probably via an increase in D-serine and D-alanine levels followed by an enhancement in NMDAR-mediated glutamatergic neurotransmission [295]. Moreover, in transgenic *Grin1^D481N^* mice displaying psychotic phenotypes, the concurrent homozygous point mutation G181R in the *DAO1* gene and the subsequent oxidase hypofunction reverted the schizophrenia-like negative and cognitive symptoms, improving spatial memory, cognitive flexibility, promoting social behaviors, and interestingly running in parallel with an increase in D-serine levels [296]. An attempt to explore in vivo DAO influence on CNS signaling pathways has been conducted using the fMRI technique, unveiling differences in brain connectivity patterns within the left putamen, the right posterior cingulate, and the left middle frontal gyri in patients with schizophrenia when split into two subgroups based on different *DAOA* genotypes [297]. In addition, differences in *DAO* genotype have been associated with alterations in the functional connectivity of the left precuneus and right posterior cingulate gyrus, both comprised in the default mode network, in patients affected by schizophrenia but not with bipolar disorder nor in healthy controls, suggesting that DAO dysfunctions may be detrimental when acting in a complex set of multiple neurobiological defects [298]. First- and second-generation antipsychotics, beyond the well-defined action on dopaminergic and serotonergic receptors, have been seen to exert an in vitro inhibitory effect on DAO activity, which should be considered as a part of the drug mechanism of action, providing further evidence to support the putative involvement of the oxidase in the pathogenesis of schizophrenia [299,300,301].

Beyond the established hypothesis of D-serine reduction and the following NMDAR hypofunction as the putative mechanism linking oxidase to psychiatric disorders, alterations in other DAO-regulated pathways might be relevant to the emergence of a molecular milieu predisposing to the development of schizophrenia. Specifically, DAO has been found to modulate the dopaminergic system by converting D-DOPA into L-DOPA and hence constituting an alternative metabolic pathway in the dopamine synthesis [302,303,304]. On the other hand, the increase in both dopaminergic VTA neurons firing rate and frontal cortex dopamine levels under DAO genetic or pharmacological inhibitory conditions has probably been due to D-serine-mediated hyperactivation of VTA neurons expressing NMDAR [305,306]. Thus, the controversial role in dopaminergic pathway regulation exerted by the oxidase needs to be better clarified in future studies focusing on DAO net effect apart from the downstream D-serine modulation.

Furthermore, an increase in peripheral DAO levels has been observed in several conditions characterized by cognitive impairment, including Alzheimer’s disease, mild cognitive impairment, and post-stroke dementia [307,308]. Even though the putative mechanism responsible for the association between DAO levels and poor performances in cognitive tasks has not been clearly established, the production of H_2_O_2_, resulting from the enzyme catalytic activity and leading to oxidative stress, has been indicated as accountable for the cognitive decline [308]. In this regard, a burst of DAO activity and the following excess in H_2_O_2_ levels may disrupt the dynamic pro- and anti-inflammatory balance in favor of the latter, promoting the establishment of a neuroinflammatory environment [309]. Considering DAO-induced reactive oxygen species production, the administration of D-amino acids alone as an augmentation strategy for patients with schizophrenia may be counterproductive, especially on long-term cognitive endpoints. Of interest, the administration of 5-chloro-benzo[d]isoxazol-3-ol (CBIO), a DAO inhibitor, showed to enhance D-serine efficacy in mice, whereas the D-amino acid alone was not effective in attenuating the pre-pulse inhibition deficit induced by MK-801, probably via reduced catabolism, higher D-serine availability, and lower oxidative stress [236,310]. Thus, only future studies addressing the effect of DAO inhibition on neuroinflammatory markers will clarify potential clinical advantages, especially in cognitive domains, coming from the combination between D-amino acids and DAO inhibitors.

In the light of multiple scientific reports assessing and supporting the hypothesis of DAO alteration as a neurobiological hallmark of schizophrenia, novel chemical compounds targeting and suppressing the oxidase activity have been developed to ameliorate psychotic symptoms (Table 2). In this regard, sodium benzoate has been the cornerstone of oxidase inhibitory drugs, showing efficacy in improving positive but not negative or cognitive symptoms in patients with schizophrenia, as assessed by a recent meta-analysis [25]. When sodium benzoate was administered to TRS patients, a significant improvement was detected in overall symptomatology and quality of life in comparison to the placebo group [311]. Furthermore, the combination between sodium benzoate and sarcosine was revealed to be efficient at ameliorating cognitive performances and global functioning in patients with chronic schizophrenia [312]. Moreover, preclinical studies have shown controversial evidence on sodium benzoate pharmacodynamic mechanism, testifying as the drug antipsychotic effect could be achieved in a murine PCP model of schizophrenia even without increasing D-serine levels [313]. Other more selective DAO inhibitory drugs are currently tested in patients with schizophrenia and among these, luvadaxistat has shown a promising improvement in cognitive functions [314,315,316]. Thus, future studies exploring the tolerability and efficacy profile in specific disease subgroups, in addition or not to other drugs enhancing the D-amino acid pathway, will shed light on the real potential of DAO inhibitors in schizophrenia and other psychotic disorders.

The relative specificity of DAO or DAOA polymorphisms in combination with brain functional connectivity alterations or other risk genetic variants for schizophrenia and the ongoing development of more selective and potent oxidase inhibitory agents may open novel possible future directions in both diagnosis and treatment of psychotic disorders, driving to a theranostic innovation in the psychiatric field too.

## 7. Other Modulators of the Glycine B Site at NMDAR

### 7.1. Glycine

Glycine is a small nonessential amino acid not containing a chiral center functioning as both an inhibitory and excitatory neurotransmitter depending on the binding site in CNS. The inhibitory action is mediated by a ligand-gated ion channel known as GlyR, pharmacologically characterized by strychnine sensitivity, and mainly localized in the spinal cord where it plays an important role in nociceptive transmission [319,320,321,322,323,324,325]. On the other hand, the excitatory effect depends on the glycine B site, defined as a modulatory strychnine-insensitive site of the GluN1 subunit at the NMDAR ion channel. In the synaptic space, glycine is released by neighboring glial cells and in small amounts by glycinergic neurons. Serine, its endogenous precursor, is converted into glycine by serine-hydroxymethyltranferase (SHMT). A driblet of glycine derives also from N-methyl-glycine (sarcosine) demethylation. A normal diet contains about 2 g of glycine and normally has no significant effect on brain levels. The glycine permeability through the blood–brain barrier (BBB) is very low after peripheral administration; therefore, high oral doses are necessary to obtain an effective increase in CNS levels [326]. The potential involvement of glycine in the modulation of other neurotransmitters systems (such as dopamine-glutamate interplay) and in the neurobiological substrates of mental disorders is supported by the association between clinical phenotypes of schizophrenia with genetic variants in glycinergic pathways and abnormal circulating levels of this molecule [327,328,329,330]. In fact, circulating levels of glycine have been found to correlate with schizophrenia symptoms [331,332]. Moreover, a negative association has been demonstrated between glycine plasma levels and pre-pulse inhibition, a measure of sensorimotor gating commonly found reduced in schizophrenia [333].

An MRS study, evaluating glutamate and glycine levels in both the anterior and posterior cingulate cortex in patients with first-episode psychosis, detected an increase in these two amino acids compared to controls, consistent with the glutamatergic dysfunctions since the acute early phase of psychotic illnesses [328].

Based on these findings, and given the NMDAR hypofunction hypothesis of schizophrenia, the ability of glycine to enhance NMDAR-mediated neurotransmission and its good tolerability profile in both acute and chronic treatment strongly suggests that this molecule might be useful as a therapeutic approach in the pharmacotherapy of schizophrenia, and possibly in TRS [150]. For this purpose, several studies were conducted testing glycine as an add-on therapy to standard antipsychotic treatment. In the literature, fourteen studies have been conducted exploring the efficacy of glycine as an augmentation strategy in schizophrenia treatment, including 11 placebo-controlled randomized clinical trials (RCT) [326,334,335,336,337,338,339,340,341,342,343] and three open-label studies [344,345,346] (Table 3). Recently, data from RCTs have been meta-analysed, showing interesting results. In particular, among NMDAR co-agonists administered in addition to canonical antipsychotics, glycine has proved to be effective in reducing SANS and PANSS total scores, as well as in treatment-refractory patients [229]. On the contrary, NMDAR modulator augmentation was not effective in patients receiving clozapine, probably due to the clozapine “ceiling” effect on the enhancement of NMDAR transmission [229,347,348,349]. These observations may account for divergent and somewhat inconsistent results of glycine augmentation efficacy in those clinical trials enrolling only patients receiving clozapine or which did not distinguish patients on clozapine from the ones on other antipsychotics. With regard to safety outcomes, patients treated with glycine experienced more dry mouth and nausea in comparison with the placebo group [229].

#### 7.1.1. Glycine Transport Inhibitors for the Treatment of Schizophrenia

**Figure 4:**
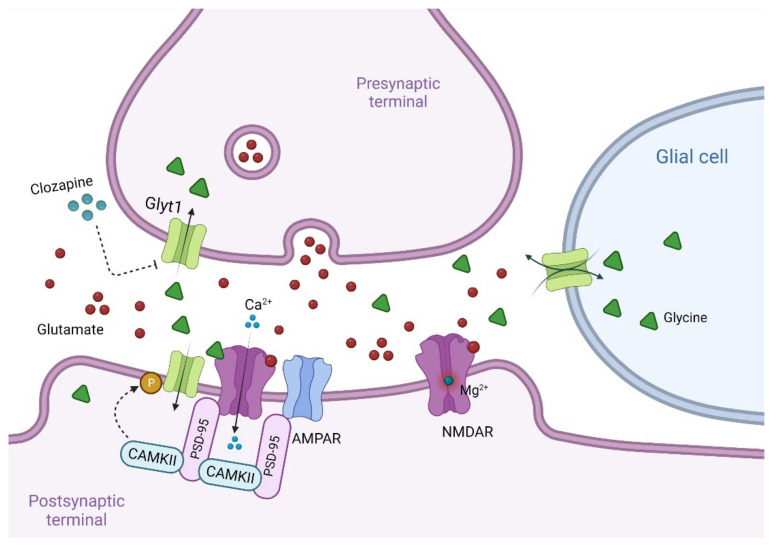
Glycine transporter 1 (GlyT1) controls glycine concentration in the synaptic cleft. GlyT1 is localized at the postsynaptic density of glutamatergic synapses and may physically interact with NMDAR complex. GlyT1 activity is regulated by indirect phosphorylation by CaMKII. It has been proposed that clozapine may exert its antipsychotic activity, among other mechanisms, by inhibiting GlyT1. GlyT1: Glycine Receptor Transporter 1; NMDAR: N-Methyl-D-aspartate receptors; PSD-95: postsynaptic density protein 95; CaMKII: Ca2+/calmodulin-dependent protein kinase; AMPAR: α-amino-3-hydroxy-5-methyl-4-isoxazolepropionic acid receptor; PSD-95: postsynaptic density protein 95. Mg^2+^: magnesium. Created with BioRender.com (accessed on 14 June 2022).

Another possible approach to improve glutamatergic neurotransmission via NMDAR would be to pharmacologically increase glycine synaptic levels by Glycine Transporter-1 (GlyT-1) inhibitors. GlyT-1 is a sodium/chloride-dependent transporter mainly expressed on glial cells and neurons, particularly on both pre- and postsynaptic terminals (Figure 4) [368,369], allowing for glycine reuptake from the synaptic cleft [370], thus maintaining subsaturating concentrations of glycine at NMDARs [371]. Multiple interactions between GlyT-1 and PSD proteins have been proposed. In particular, PSD-95 stabilizes and anchors GlyT1 at the plasma membranes [369], whereas several kinases, including the Ca^2+/^calmodulin-dependent protein kinase, phosphorylate the cytosolic regions of GlyT-1, regulating its activity [372].

The importance of GlyT-1 has been proven in preclinical studies conducted in GlyT-1 knock-out mice. In fact, the absence of the GlyT-1 gene has been found to lead to severe respiratory and motor deficits causing premature death [373]. Moreover, GlyT-1 deletion has been associated with neonatal encephalopathy in humans, characterized by impaired consciousness, unresponsiveness, absence of reflexes, hypotonia, and respiratory failure [374]. However, heterozygous GlyT1^+/−^ mice, associated with a reduced but not null activity of GlyT-1, displayed increased memory retention [330] and a distinctive resistance to psychotogenic effects of amphetamines [375].

Given that glycine transport maintains local synaptic glycine concentration at very low levels [376], we can assume that GlyT-1 activity might be targeted in schizophrenia, in order to mitigate the NMDAR hypofunction, reaching a fine-tuning of the excitation-inhibition balance.

Among GlyT-1 inhibitors, sarcosine and bitopertin were tested as adjunctive therapy for schizophrenia (Table 2), as discussed in the sections below.

#### 7.1.2. Sarcosine

Sarcosine, also known as N-methylglycine, is a degradation product of the amino acid glycine. Preclinical studies supported by in vivo brain neuroimaging demonstrated that sarcosine acts as a potent and selective GlyT-1 inhibitor, thus potentiating NMDAR functions and enhancing hippocampal LTP [377], suggesting that this compound may exert antipsychotic and precognitive effects [378,379]. Sarcosine mechanism of action might involve the specific synaptic elevation of glycine but not D-serine, as suggested by microdialysis findings in the PFC [380]. It has been proposed that, given the structural similarity to glycine, sarcosine may directly act also as an NMDAR co-agonist [381] and inhibitory glycine receptor ligand, even less potent than glycine [382]. In fact, sarcosine has been found to elicit the same effects as glycine on NMDAR activation, even in the absence of glycine itself, probably directly binding to the glycine B site [381].

As observed with other D-amino acids, the combination with clozapine does not appear to be effective, probably due to the glycinergic action intrinsically exerted by clozapine, currently the only antipsychotic indicated and evidence-based treatment for TRS [3]. Moreover, sarcosine administration induced a pattern of c-Fos expression resembling that induced by clozapine [377]. Therefore, sarcosine and other D-amino acid-centered strategies may represent alternatives to clozapine in refractory patients.

Several studies investigated sarcosine augmentation efficacy in schizophrenia patients [224,225,312,347,350,352,353,354]. The addition of sarcosine (2 g/day) to antipsychotic medications, including risperidone [350] and other atypicals [225] but not clozapine [347], was found to benefit schizophrenia patients. A randomized double-blind placebo-controlled study demonstrated that sarcosine (2 g/day) is more effective in reducing psychotic symptoms than placebo or D-serine (2 g/day) [224,347]. Moreover, a randomized double-blind study reported that sarcosine alone at the dose of 2 g/day was useful in the treatment of acutely symptomatic drug-free patients affected by schizophrenia [351], suggesting that sarcosine can benefit not only chronic patients but also acutely ill subjects. Furthermore, the addition of sarcosine (2 g/day) has been reported to positively affect the glutamatergic transmission by reducing Glx (a complex of glutamate, glutamine, and GABA)/creatine ratio in white matter of the left frontal lobe as well as in the hippocampus [353,354].

However, these studies have been based on a small sample size of patients, so further large-size placebo-controlled dose finding trials are needed to fully understand the role of sarcosine in schizophrenia. A recent meta-analysis showed that sarcosine surpassed non-sarcosine GlyT-1 inhibitors (i.e., bitopertin) as an augmentation strategy in head-to-head comparisons, mainly targeting negative symptoms [229].

According to this evidence, novel selective GlyT-1 inhibitors have been developed and evaluated for the treatment of schizophrenia. Gaining a more complete understanding of GlyT-1 role in the treatment of schizophrenia patients is expected to provide new therapeutic perspectives for managing this disabling disorder.

#### 7.1.3. Non-Sarcosine Derivatives GlyT-1 Inhibitors

Preclinical studies investigating the effects of sarcosine derivates in rodents have shown the occurrence of numerous side effects such as ataxia, motor and respiratory dysfunctions [327], probably related to the slow dissociation kinetics that allow sarcosine-derivatives to act as pseudo-irreversible inhibitors of GlyT-1 [383]. In this framework, pharmaceutical companies decided to develop non-sarcosine derivatives GlyT-1 inhibitors, such as bitopertin, also known as RG1678 or RO4917838.

Regarding bitopertin, preclinical studies showed its ability to modulate both glutamatergic and dopaminergic signaling, enhance hippocampal LTP [377], and improve performance in PFC-dependent tasks [384], thus mitigating schizophrenia-like behaviors. Early clinical studies pointed to the efficacy of adjunctive bitopertin in treating negative symptoms [355,384], although an inverted U-shaped dose–response profile emerged, leading to inconsistent results when bitopertin was administered at high doses (=60 mg). These results were paralleled, at the preclinical level, by the observations that, at higher concentrations, bitopertin did not affect LTP induction processes, probably as a result of internalization of the NMDAR following an excessive release of glycine [327]. However, in a phase III 24-week double-blind placebo-controlled study, bitopertin failed to reach the primary and secondary endpoints in patients with persistent predominant negative symptoms [356], and further clinical trials testing this molecule were discontinued by Roche. Kantrowitz et al. reported that bitopertin, even at the dose that was most effective in the clinical development programs (10 mg), did not affect NMDAR-based biomarkers including auditory mismatch negativity and visual P1, neurophysiological measures indexing local circuit dysfunctions involved in altered sensory processing and cognitive functioning [357].

Though the bitopertin mechanism of action appeared to be promising, the inverted U-shaped dose–response curve, associated with the wide range of inter-individual variability, may have complicated the identification of the dose needed for efficacy in humans.

Another non-sarcosine GlyT-1 inhibitor, BI 425809, has been recently tested in a double-blind randomized controlled phase II study in patients affected by schizophrenia, resulting in improvements in cognitive symptoms, as assessed by Measurement and Treatment Research to Improve Cognition in Schizophrenia (MATRICS) cognitive battery [358]. The study investigated the effects of 2 mg, 5 mg, 10 mg, or 25 mg BI 425809 administered daily for 12 weeks, as an add-on strategy to antipsychotic monotherapy and cotreatment with a second antipsychotic [358]. BI 425809 was well tolerated in healthy volunteers as well as in patients [358,385]. A randomized trial combining BI 425809 with computerized cognitive training has been recently planned in order to consolidate the results obtained on cognitive functions [384].

### 7.2. D-Cycloserine

D-cycloserine is a cyclic analogue of serine, which, due to its ability to inhibit the bacterial D-amino acid transaminase, has been marketed as a second-line antibiotic in tuberculosis treatment [386]. Given its action as a partial agonist at the glycine B site, it has been tested in clinical trials as an adjuvant treatment for anxiety disorders, depression, trauma-related, obsessive compulsive disorder [387,388,389,390,391,392], and schizophrenia [388,393]. Regarding D-cycloserine effects in psychiatric disorders, a preclinical study has investigated its role in the modulation of Homer 1a expression after typical and atypical acute antipsychotics administration, showing that the haloperidol and clozapine effect of increasing Homer1a levels in the caudate-putamen and nucleus accumbens was attenuated by D-cycloserine co-administration [394]. D-cycloserine, reducing Homer1a expression and thus its “negative dominant” effect, may enhance mGluR clustering, resulting in a modulation of neuronal and postsynaptic plasticity that might be implicated in the antipsychotic mechanism of action [112,394].

In line with these preclinical observations, several RCTs have been conducted to explore the potential of D-cycloserine as an add-on to antipsychotic treatment for schizophrenia [343,359,360,361,362,363,364,365,366,367,395] but two recent meta-analyses agreed that D-cycloserine failed to exhibit significant efficacy in treating negative, cognitive, or positive symptoms of schizophrenia [229,396].

### 7.3. D-Peptides

Several D-peptides have recently emerged as potential therapeutic candidates for Alzheimer’s disease and other neurological conditions [397,398]. D-peptides, synthetized in D-enantiomer form by mirror image phage display methodology, are designed to extend the half-life of the peptides and increase resistance to enzyme degradation, prolonging in vivo activity [399]. In particular, D-enantiomeric forms of L-peptides interact with the target proteins of the natural L-amino acid configuration. This technique was employed to develop Tau aggregation inhibitors [398,400] as well as promising D-Aβ-peptides to reduce plaque formation and inflammatory reactions [401,402,403] in Alzheimer’s disease.

Despite limited clinical evidence, D-peptide therapeutics is a rapidly expanding field [404] offering multiple advantages, including low-cost synthesis, low immunogenicity, and serum stability compared to L-peptides analogues that are faster degraded in serum by active endopeptidase. Thus, D-peptides are currently designed and tested in preclinical research setting for a variety of medical conditions, including infections and cancer [405,406,407].

## 8. Discussion

In the last twenty years, the difficulty of explaining the complexity of schizophrenia clinical presentation by a single brain neurochemical abnormality or one synaptic molecular alteration, as well as the evidence of multiple patterns of connectivity derangements, have raised a keen interest in innovative pharmacological treatments targeting different hallmarks of the disorder, in the attempt to ameliorate the multidomain psychopathological manifestations and provide novel alternatives for TRS patients [1,13,406,407,408,409,410,411,412,413].

In this framework, among multiple strategies proposed to bypass the difficulties of TRS treatment, especially in patients not eligible or non-responder to clozapine, D-amino acids (i.e., D-serine, D-alanine, and D-aspartate) and related proxy molecules (e.g., D-cycloserine, sarcosine) have attracted interest as potential innovative therapies [414]. All D-amino acids and proxy molecules clinically tested have been explored usually as add-on/augmentation therapy or in protocols including patients with poor response to antipsychotics [150], showing a significant capability in decreasing the severity of the overall or subsets of symptoms in schizophrenia and TRS groups [229], even though several limitations of the trials should be acknowledged.

First, the number of patients included in each single study is low, and even if coherent with a pilot study, there is a need for trials with larger sample sizes in order to draw firmer conclusions.

Second, single trials are not easily comparable even among studies investigating the same compound due to different study designs adopted by clinicians, specifically exploring disparate populations and using different antipsychotics when the D-amino acid is tested as add-on therapy [222,224,311,312,318].

Third, all the available D-amino acids tested in schizophrenia and TRS patients are given orally at high doses to ensure significant adsorption and even more important reliable brain levels of the molecules. This is a crucial issue: at present, due to the lack of imaging strategies as the ones that can mirror the quantification of dopamine receptors occupancy by antipsychotics, it is impossible to ascertain with precision, which is the in vivo pharmacodynamics of D-amino acids, hindering the search for the optimal dose accordingly.

Fourth, the cluster of symptoms targeted by the treatment with D-amino acids appears to be different in different studies and complicates the overall comparative interpretation of the studies’ outcomes.

Despite these limitations, the search for a “D-aminoacidergic” compound is strongly pursued both by academia and industry.

In this regard, beyond the molecules extensively discussed above, an emerging field is represented by the investigation of D-cysteine function in the brain and its possible involvement in the pathophysiology and treatment of behavioral disorders. Specifically, D-cysteine binds to Myristoylated Alanine Rich C Kinase Substrate (MARCKS), which is relevant for neuronal survival and migration via Akt pathway regulation [415]. Akt dysfunction has been associated with abnormal dopamine–glutamate interaction in schizophrenia [416,417,418,419] and is reported to be involved in the mechanism of action of antipsychotics [420,421]. The augmentation of the antipsychotic therapy with D-cysteine in TRS could represent an innovative strategy deserving attention and worth being explored in pilot clinical studies, also considering the available information regarding the role of L-cysteine and acetylcysteine in the treatment of behavioral disorders [422].

Moreover, D-aspartate has been well-characterized in mammalian brains [24], and multiple findings from in vivo and in vitro preclinical studies as well as post-mortem investigations in patients have highlighted the possible association with schizophrenia pathophysiology and the putative translational value of this D-amino acid in schizophrenia treatment. In this regard, two findings are worth considering: (1) D-aspartate levels have been found increased after olanzapine chronic administration in mice possibly via the DASPO inhibition, which may represent an unveiled antipsychotic mechanism of action [282] (2) DASPO is responsible for a stable control on D-aspartate concentrations in the brain, differently from DAO, whose low activity affects D-serine concentrations only to a lesser extent [271,272]. Based on the NMDAR dysfunction hypothesis, it has been proposed that inhibitors of the human D-aspartate oxidase (hDASPO) [24] could represent a potentially innovative strategy to counteract the loss of response to antipsychotics in schizophrenia.

The search for “D-aminoacidergic” compounds is an emerging field with significant room for further expansion and few initial results are at the present under evaluation: sodium benzoate (NaBen) for TRS and TAK-831 (luvadaxistat) potentially for negative symptoms of schizophrenia are two examples of a pharmacological strategy based on D-amino acid indirect modulation [309,310,314]. The discovery of a novel class of DAO inhibitors with the Schrödinger computational platform needs to be followed closely in the next few years to establish the real possibility of a clinical application [314].

Regarding possible future directions in clinical and preclinical research, in the light of previous findings extensively discussed above, D-amino-acid-centered strategies might represent an alternative rather than an augmentation approach to clozapine in TRS patients due to a putative “ceiling” effect on NMDAR neurotransmission. In addition, to counteract the possible detrimental effects of high doses of D-amino acids, co-administration of DAO inhibitors might represent a novel therapeutic strategy as testified by preclinical models [236], exploiting the concomitant benefits of complementary molecular pathways, and minimizing possible adverse effects. Furthermore, the development of specific DAO ligands for PET imaging studies, overcoming the problem of BBB low permeability [423], might provide a novel in vivo perspective and serve as a new line of research, clarifying the impact of enzyme changes under multiple pathophysiological conditions, including TRS. The ongoing fine-tuning of genetic and brain imaging techniques, combined with computational approaches, will implement current knowledge on D-amino acids and related enzymes, shedding light on their relevance for TRS neurobiology and providing novel hallmarks of the disease. In this regard, the “D-aminoacidergic” pathway might drive a theranostic innovation, offering both diagnostic and therapeutic targets in TRS.

In conclusion, TRS represents a relevant epidemiological burden affecting almost 30% of schizophrenia patients and significantly impacting the life of the subjects in terms of reduced functioning, cognitive impairment, and overall quality. Only one pharmacological treatment almost fifty years after its first introduction, clozapine, is still available at the present for TRS. Therefore, the search for better and innovative pharmacological strategies is needed, and direct and reverse translational implications as well as preliminary clinical evidence support the D-amino acids strategy for the development of novel and safer therapeutic agents worth exploration in clinical trials.

## Figures and Tables

**Table 1:** Randomized clinical trials investigating the efficacy of D-amino acids in treating schizophrenia.

Agent Added	Author	Study Design	Groups of Treatment	Mean Age	n	Dose	Duration	Stable Antipsychotic Regimens		Outcome	Side Effects
**D-serine**	Kantrowitz et al., 2018 [223]	Double-blind crossover trial	D-serine	40 ± 11	14	60 mg/kg/day	6 weeks	CPZ equivalents (mg): 965 ± 760		Improvement in total PANSS symptoms Reduction in evoked power for the α band	-
			Placebo			-					
	Weiser et al., 2012 [228]	RCT	D-serine	Not retrieved	195	2 g/day	-	Not retrieved		No significant differences in SANS and MATRICS battery score	-
			Placebo								
	Tsai et al., 1998 [226]	Double-blind, placebo-controlled trial	D-serine	31.7 ± 7.5	15	30 mg/kg/day	6 weeks	Sulpiride (n = 6), Haloperidol (n = 5); Risperidone (n = 4); Pipotiazine (n = 2) Fluphenthixol (n = 2) Thiothixene (n = 1) Pipotiazine (n = 2) Haloperidol and pipotiazine (n = 1) Pipotiazine and chlorpromazine (n = 1) Fluphenazine and chlorpromazine (n = 1) Risperidone and chlorpromazine (n = 1) Trifluoperazine and chlorpromazine (n = 1) Trifluoperazine and haloperidol (n = 1) One patient was antipsychotic free.		Improvement in PANSS positive SANS PANSS-cognitive WCST CGI	No significant side effects
			Placebo	33.9 ± 6.6	14	-					
	Heresco-Levy et al., 2005 [222]	double-blind, placebo-controlled trial	Risperidone + D-serine Olanzapine + D-serine	42.7 ± 13.1	21	30 mg/kg/day	6 weeks	-		Improvement in BPRS SANS PANSS (negative, positive, cognitive)	No significant clinical or laboratory side effects
			Risperidone + Placebo Olanzapine + Placebo	47.4 ± 14.2	18						
	Lane et al., 2005 [224]	RCT	D-serine	31.8 ± 10.2	21	2 g/day	6 weeks	Risperidone		No significant differences	Weight gain (67%) Palpitation (42%)
			Placebo	34.1 ± 8.7	23						
	Lane et al., 2010 [225]	RCT	D-serine	30.7 ± 9.6	20	2 g/day	6 weeks	Risperidone Olanzapine Quetiapine		No significant differences	No significant side effects
			Placebo	31.5 ± 7.9	20						
	Tsai et al., 2010 [227]	RCT	D-serine	42.6 ± 3.6	10	30 mg/kg/day	6 weeks	Clozapine mean dose: 363 ± 128		No significant differences	No differences in side effects
			Placebo	39.5 ± 5.5	10			Clozapine mean dose: 315 ± 146			
	Kantrowitz et al., 2010 [230]	Open label trial	D-serine low dose	41.7 ± 11.4	12	30 mg/kg		CPZ Equivalents/day	468.8 ± 252	Improvement in PANSS and CGI	-
			D-serine mild dose	43.5 ± 9.4	19	60 mg/kg			602.6 ± 295		-
			D-serine high dose	43.2 ± 9.6	16	120 mg/kg			493.7 ± 319		Nephrotoxic-like pattern at 120 mg/kg which resolved upon D-serine discontinuation (6.25%) Asymptomatic transaminitis (12.5%) Insomnia after one dose and GI distress (12.5%)
	Kantrowitz et al., 2015 [26]	Double-blind, placebo-controlled trial	D-serine	13–35	15	60 mg/kg/day	16 weeks	-		Scale of Prodromal Symptoms negative score	-
			Placebo		20	-					
**D-alanine**	Tsai et al., 2006 [27]	RCT	D-alanine	30.9 ± 6.5	14	100 mg/kg daily	6 weeks	CPZ equivalents (mg): 468 ± 478	Sulpiride; Haloperidol; Risperidone; Pipotiazine; Pipotiazine and chlorpromazine; Pipotiazine and sulpiride; Thioridazine; Trifluoperazine and chlorpromazine; Fluphenazine decanoate and chlorpromazine	Improvement in PANSS-positive PANSS-cognitive and SANS score	No significant differences
			Placebo	31.8 ± 7.4	18			CPZ equivalents (mg): 364 ± 220			

PANSS, Positive and Negative Syndrome Scale; SANS, Scale for the Assessment of Negative Symptoms; MATRICS, Measurement and Treatment Research to Improve Cognition in Schizophrenia; WCST, Wisconsin Card Sorting Test; CGI, Clinical Global Impression; BPRS, Brief Psychiatric Rating Scale; RCT, Randomized Controlled Trial; TRS, Treatment-Resistant Schizophrenia; CPZ, chlorpromazine.

**Table 2:** Randomized clinical trials investigating the efficacy of sodium benzoate as a DAO-inhibitory agent in treating chronic schizophrenia and early psychosis.

Authors	Groups of Treatment	Mean Age	n	Dose	Duration	Stable Antipsychotic Regimens	Outcome	Side Effects
Lane et al., 2013 [317]	Sodium benzoate	38.4 ± 9.7	25	1 g/day	6 weeks	Amisulpride Chlorpromazine Flupenthixol Haloperidol Quetiapine fumarate Risperidone Sulpiride Ziprasidone Zotepine	The sodium benzoate group exhibited better performances in SANS, GAF, QOLS, CGI, HDRS, speed of processing, visual learning and memory	No significant differences were found between the two groups in Simpson-Angus Rating Scale, AIMS, and Barnes Akathisia Scale scores
	Placebo	36.3 ± 7.9	27	-				
Scott et al., 2020 [318]	Sodium benzoate	21.7 ± 4.7	49	1 g/day	12 weeks	Aripiprazole Amisulpride Brexpiprazole Clozapine Haloperidol Olanzapine Lurasidone Paliperidone Quetiapine Risperidone	No improvements were found in the sodium benzoate group	No significant differences were found between groups
	Placebo	21.2 ± 3.4	50	-				
Lin et al., 2018 [311]	Sodium benzoate	44.3 ± 7.2	20	1 g/day	6 weeks	Clozapine	Improvements in negative and overall symptomatology and quality of life were detected in sodium benzoate groups compared to placebo	No significant differences were found between the two groups in Simpson-Angus Rating Scale, AIMS, and Barnes Akathisia Scale scores
	Sodium benzoate	44.8 ± 8.1	20	2 g/day				
	Placebo	47 ± 11.9	20	-				
Lin et al., 2017 [312]	Sarcosine + sodium benzoate	37.8 ± 9.6	21	2 g/day + 1 g/day	12 weeks	Amisulpride Aripiprazole Olanzapine Paliperidone Quetiapine Risperidone Zotepine	Improvement in GAF and cognitive battery compared to sarcosine group	-
	Sarcosine	38.2 ± 9.3	21	2 g/day			Improvement in reasoning and problem solving compared to placebo	
	Placebo	39.1 ± 9.5	21	-			-	

AIMS, Abnormal Involuntary Movement Scale; CGI, Clinical Global Impression; GAF, Global Assessment of Function; HDRS, Hamilton Depression Rating Scale–17 items; QOLS, Quality of Life Scale; SANS, Scales for the Assessment of Negative Symptoms–20 item; RCT, randomized controlled trial.

**Table 3:** Randomized clinical trials investigating the efficacy of glycine-centered agents in treating schizophrenia.

Agent Added	Author	Study Design	Groups of Treatment	Mean Age	n	Doses	Duration	Stable Antipsychotic Regimens	Outcome	Side Effects
**Glycine**	Serrita et al., 2019 [334]	RCT	Glycine	48.60 ± 5.01	10	0.8 g/kg	12 weeks	Antipsychotics	No significant differences in PANSS score	No significant differences
			Placebo	49.20 ± 4.84	10					
	Rosse et al., 1989 [345]	Open-label Pilot study	Glycine	38 ± 15	6	18.8 g/day	8 weeks	Haloperidol; Benztropine; Thiothixene; Vitamin E; Loxapine	Beneficial effects in two patients at SANS scale. Two others are worsened	Reduction in neuroleptic-induced muscle stiffness and extrapyramidal dysfunction in three patients.
	Potkin et al., 1999 [335]	RCT	Glycine	12.4 ± 7.2	9	30 mg/day	12 weeks	Clozapine (400–1200 mg/day)	No significant treatment effects	No significant differences
			Placebo		10				Improvement in BPRS positive symptoms	
	Javitt et al., 1994 [336]	RCT	Glycine	-	14	30 mg/day	8 weeks	Unknown	Improvement in PANSS negative symptoms domain	-
			Placebo							
	Heresco-Levy et al., 1999 [339]	RCT	Glycine	38.8 ± 11.0	9	61.2 ± 13.4	6 weeks	Clozapine mean dose: 471.0 ± 207.8 mg Chlorpromazine equivalents: 240.0 ± 146.3	Significant increase in glycine and serine levels Significant improvement in PANSS—negative symptoms and BPRS total score	No significant differences
			Placebo		10					
	Buchanan et al., 2007 [343]	RCT	Glycine	42.6 ± 10.8	42	60 g/day	16 weeks	Unknown	non-significant differences in SANS score Significant improvement in CGI score in D-cycloserine group compared to placebo	Worsened nausea and dry mouth in glycine group compared to placebo
			D-cycloserine	44.4 ± 10.4	46	50 g/day				
			Placebo	43.4 ± 11.4	45	-				
	Costa et al., 1990 [344]	Open-label Pilot study	Glycine	34.8	11	15 g/day	5 weeks	Increasing amount in second generation antipsychotics	Decrease in BPRS score in 33% of patients	Upper gastrointestinal discomfort in 17% of patients
	Liederman et al., 1996 [346]	Open-label Pilot study	Glycine	45.0 ± 7.6	5	60.0 g/day	8 weeks	Clozapine (2) Risperidone (2) Haloperidol (1)	Significant improvement in negative symptoms was found using the SANS	No adverse effects; Reduction in EPS
	Heresco-Levy et al., 1996 [338]	RCT	Glycine	41.36 ± 12.93	11	60.63 ± 12.98	6 weeks	Clozapine (4) Thioridazine (3) Haloperidol (2) Chlorpromazine (1) Perphenazine (1)	Improvement in PANSS-negative symptoms PANSS-general psychopathology and PANSS total score	No adverse effects
			Placebo							
	Javitt et al., 2001 [337]	RCT	Glycine	39.6 ± 5.5	12	0.8 g/kg/day	6 weeks	Olanzapine (6) Clozapine (4) Mesoridiazine (1) Haloperidol (1)	Improvement in PANSS- negative symptoms	No significant adverse effects
								Unknown		
			Placebo							
	Evins et al., 2000 [341]	RCT	Glycine	39.0 ± 7.0	27	60 g/day	8 weeks	Clozapine	No significant treatment effects	No significant adverse effects
			Placebo							
	Diaz et al., 2005 [342]	RCT	Glycine	39.5 ± 12.44	6	60 g/day	8 weeks	Clozapine mean dose: 575 mg/day	No significant treatment effects	Nausea and vomiting (16%)
			Placebo		6					-
	Greenwood et al., 2018 [340]	RCT	Glycine	36.0 ± 7.8	12	24.8 g/day	6 weeks	Unspecified antipsychotics medication	Improvement in PANSS-Total, PANSS-Negative and PANSS-General symptoms Improvement in MMN	-
			Placebo	40.2 ± 8.9	10					
	Heresco-Levy et al., 2004 [326]	RCT	Glycine	44.7 ± 10.8	7	55.2 ± 10.1 g/day	6 weeks	Olanzapine (10) mean dose: 14.3 ± 6.2 mg/day Risperidone mean dose (4): 6.20 ± 3.08 mg/day	Improvement in five-factor PANSS	Decrease in EPS, upper gastrointestinal tract discomfort with nausea (12%)
			Placebo		7					
**Sarcosine**	Lane et al., 2005b [224]	RCT	Sarcosine	36.1 ± 10.2	21	2 g/day	6 weeks	Risperidone	Improvement in PANSS total, positive, and negative score; SANS-20 and SANS-17	Treatment-emergent adverse events other than extrapyramidal symptoms are similar in the three groups
			D-serine	31.8 ± 10.4	21	2 g/day			-	
			Placebo	34.1 ± 8.7	23	-			-	
	Lane et al., 2006 [347]	RCT	Sarcosine	36.7 ± 10.1	10	2 g/day	6 weeks	Clozapine mean dose: 306 ± 159 mg	Non-significant differences	Non-significant differences
			Placebo	35.5 ± 6.6	10			Clozapine mean dose: 305 ± 55 mg		
	Lane et al., 2010 [225]	RCT	Sarcosine	30.4 ± 10.6	20	Not retrieved	6 weeks	Risperidone Olanzapine Quetiapine	Not retrieved	-
			Placebo	31.5 ± 7.9	20					
			D-serine	30.7 ± 9.6	20					
	Lin et al., 2017 [312]	RCT	Sarcosine + sodium benzoate	37.8 ± 9.6	21	2 g/day + 1 g/day	12 weeks	Amisulpride Aripiprazole Olanzapine Paliperidone Quetiapine Risperidone Zotepine	Improvement in GAF and cognitive battery compared to sarcosine group	-
			Sarcosine	38.2 ± 9.3	21	2 g/day			Improvement in reasoning and problem solving compared to placebo	
			Placebo	39.1 ± 9.5	21	-			-	
	Tsai et al., 2004 [350]	RCT	Sarcosine	29.8 ± 7.2	17	2 g/day	6 weeks	Chlorpromazine equivalence: 409 ± 320	Improvement in PANSS score	No significant side effects
			Placebo	33.4 ± 8.3	21			Chlorpromazine equivalence: 433 ± 243		
	Lane et al., 2008 [351]	RCT	Sarcosine	31.3 ± 10.4	9	1 g/day	6 weeks	Drug-free	No significant differences between groups	Insomnia, weight gain (1-2 kg), sedation, constipation, and fatigability. These effects were all mild and brief.
			Sarcosine	34.3 ± 11.2	11	2 g/day				
	Strzelecki et al., 2018 [352]	RCT	Sarcosine	37.3 ± 11.3	29	2 g/day	6 months	First- and second-generation antipsychotics	Improvement in total, general, and negative PANSS scores	No significant side effects
			Placebo	40.2 ± 10.1	30					
	Strzelecki et al., 2015 [353,354]	RCT	Sarcosine	36.5	25	2 g/day	6 months	Antipsychotics except clozapine	Improvement in total PANSS scores	No significant side effects
			Placebo	40	25					
**Bitopertin**	Umbricht et al., 2014 [355]	RCT	Bitopertin high dose	38.9 ± 9.5	79	60 mg/day	8 weeks	Aripiprazole Olanzapine Quetiapine Paliperidone Risperidone Risperidone long-acting injection	-	Serious adverse effects in 3.8% of patients
			Bitopertin mild dose	40.7 ± 9.4	81	30 mg/day			Improvement in PANSS and NSFS score	Serious adverse effects in 2.4% of patients
			Bitopertin low dose	41.1 ± 10.4	82	10 mg/day			Improvement in PANSS, NSFS, CGI-I-N, and PSP score	Serious adverse effects in 1.21% of patients
			Placebo	30.0 ± 10.8	81				-	-
	Bugarski-Kirola et al., 2016 [356]	RCT	Bitopertin high dose	39.1 ± 12.2	202	20 mg/day	12 weeks	Unspecified	-	1%
			Bitopertin low dose	40.2 ± 12.4	200	10 mg/day			Improvement in PANSS and PSFS score compared to placebo	2% three deaths due to upper gastrointestinal hemorrhage caused by duodenal ulcer, alcohol poisoning and related head injury, and suicide
			Placebo	39.7 ± 12.7	200	-			-	3.5%
	Kantrowitz et al., 2017 [357]	RCT	Bitopertin	40 ± 11	17	10 mg/day	6 weeks	CPZ equivalents > 600 mg	No differences between groups	No side effect
			Placebo	43 ± 12	12	-				
**BI425809**	Fleischhacker et al., 2021 [358]	RCT	BI425809	36.5 ± 8.5	85	2 mg/day	12 weeks	Unspecified antipsychotics treatment	Improvement in cognitive functions in BI425809 groups compared to placebo. The largest enhancement from baseline vs. placebo was observed in the 10 mg and 25 mg dose group	Headache Somnolence Gastrointestinal disorders
			BI425809	37.5 ± 7.9	84	5 mg/day				
			BI425809	37.9 ± 6.8	85	10 mg/day				
			BI425809	36.2 ± 7.8	85	25 mg/day				
			Placebo	37.2 ± 7.7	170	-				
**D-cycloserine**	Cain et al., 2014 [359]	RCT	D-cycloserine	48.8 ± 11.5	18	50 mg/day	8 weeks	Clozapine and others (unspecified)	Improvement in performance on the auditory discrimination task, working memory, and negative symptoms	-
			Placebo	46.2 ± 13.3	18					
	Duncan et al., 2004 [360]	RCT	D-cycloserine	48.7 ± 12.1	10	250 mg/day	4 weeks	Unspecified	Improvement in SANS and BPRS score	No side effects
			Placebo	54.4 ± 11.8	12					
	Goff et al., 1999 [361]	RCT	D-cycloserine	36.6 ± 9.6	17	50 mg/day	6 weeks	Unspecified	Worsening in negative symptoms	-
			Placebo							
	Goff et al., 1999 [362]	RCT	D-cycloserine	46.8 ± 12.3	23	50 mg/day	8 weeks	Unspecified	Improvement in SANS score	-
			Placebo	41.2 ± 8.1	24					
	Goff et al., 2005 [363]	RCT	D-cycloserine	45.9 ± 7.4	22	50 mg/day	6 months	Unspecified	No significant differences	9%
			Placebo	47.0 ± 8.6	28					21%
	Goff et al., 2008 [364]	RCT	D-cycloserine	50.1 ± 9.15	19	50 mg/day	8 weeks	All antipsychotic except for clozapine	Improvement in SANS score	No side effects
			Placebo	48.0 ± 6.66	19					
	Heresco-Levy et al., 2002 [365]	RCT	D-cycloserine	40.0 ± 12.1	8	50 mg/day	6 weeks	Conventional antipsychotics and olanzapine, or risperidone	Improvement in SANS and PANSS negative score	Worsening of psychotic symptoms in two patients on treatment and two during placebo
			Placebo		8					
	Rosse et al., 1996 [366]	Open-label study	D-cycloserine high dose	38.1 ± 6.8	6	30 mg/day	4 weeks	Molindone 150 mg/day	No significant differences	No side effects
			D-cycloserine low dose		3	10 mg/day				
			Placebo		4	-				
	Takiguchi et al., 2017 [367]	RCT crossover study	D-cycloserine	44.0 ± 14.0	36	50 mg/day	6 weeks	Unspecified: 827.0 ± 609.5 CPZ equivalents	No significant differences	Liver enzyme elevation in two patients of the placebo group
			Placebo							

PANSS, Positive and Negative Syndrome Scale; SANS, Scale for the Assessment of Negative Symptoms; CGI, Clinical Global Impression; CGI-I-N, CGI Improvement of Negative Symptoms; GAF, Global Assessment of Function; BPRS, Brief Psychiatric Rating Scale; MMN, Mismatch negativity; NFSF, Need Satisfaction and Frustration Scale; RCT, Randomized Controlled Trial; PSP, Personal Social Performance; PSFS, Patient Specific Functional Scale; CPZ, chlorpromazine; EPS, extrapyramidal symptoms.

## Data Availability

All data are available upon request.
